# Crystallization of Random Metallocene-Catalyzed Propylene-Based Copolymers with Ethylene and 1-Hexene on Rapid Cooling

**DOI:** 10.3390/polym13132091

**Published:** 2021-06-25

**Authors:** Daniela Mileva, Jingbo Wang, René Androsch, Katalee Jariyavidyanont, Markus Gahleitner, Klaus Bernreitner

**Affiliations:** 1 Borealis Polyolefine GmbH, Innovation Headquarters, Sankt Peterstrasse 25, 4021 Linz, Austria; Jingbo.Wang@borealisgroup.com (J.W.); markus.gahleitner@borealisgroup.com (M.G.); klaus.bernreitner@borealisgroup.com (K.B.); 2 Interdisciplinary Center for Transfer-Oriented Research in Natural Sciences, Martin Luther University Halle-Wittenberg, 06099 Halle/Saale, Germany; rene.androsch@iw.uni-halle.de (R.A.); katalee.jariyavidyanont@iw.uni-halle.de (K.J.)

**Keywords:** polypropylene, random propylene copolymer, crystallization, crystal nucleation, fast scanning chip calorimetry (FSC)

## Abstract

Propylene-based random copolymers with either ethylene or 1-hexene as comonomer, produced using a metallocene catalyst, were studied regarding their crystallization behaviors, with a focus on rapid cooling. To get an impression of processing effects, fast scanning chip calorimetry (FSC) was used in addition to the characterization of the mechanical performance. When comparing the comonomer type and the relation to commercial grades based on Ziegler–Natta-type catalysts, both an interaction with the catalyst-related regio-defects and a significant difference between ethylene and 1-hexene was observed. A soluble-type nucleating agent was found to modify the behavior, but to an increasingly lesser degree at high cooling rates.

## 1. Introduction

In the large family of copolymers of isotactic polypropylene (iPP), which have been essential for the global success of this polymer with a global production volume of more than 60 million tons per year [1], random copolymers have a special position. In contrast to heterophasic copolymers [2], which are often based on an iPP homopolymer matrix and elastomeric inclusions, random copolymers derive their properties from single-component structures. The crystallinity is reduced in relation to iPP homopolymers, resulting from chain disturbances by the comonomer incorporation into the chains. This enhances the inherent polymorphism of iPP, which can crystallize not only in the predominant α-modification [3], but also the more ductile “frustrated” β-form [4], the alternately layered γ-form [5], the more recently discovered trigonal δ-form [6], the disturbed ε-form [7] and the conformationally disordered mesomorphic form [8]. Each of these forms has specific connections to the chain structure and nucleation, and several modifications offer specific application properties [9].

The type, amount and distribution of disturbances in the iPP chain result from variations of the used catalyst, comonomer type and synthesis process. The commercially most widely used heterogeneous Ziegler–Natta-type catalyst (ZNC) generates chains with some stereo-defects, with isotacticities in the range from 94% to 99%, but hardly any regio-defects [10]. This means that variations of the melting point (*T*_m_) and the maximum achievable crystallinity of ZNC-based iPP homopolymers are rather limited; for example, *T*_m_ may vary between 161 and 167 °C. In contrast to that, single-site catalysts (SSCs), of which bridged metallocenes are used for iPP polymerization, also introduce regio-defects like 1,2- and 2,1-misinsertions, resulting in a much wider variation of *T*_m_ (i.e., down to 150 °C) and of the crystallinity [11].

Further variations result from the comonomer type. Often ethylene is used [12], which is most economical but limited in amount due to the high reactivity, especially with ZNC. At rather moderate ethylene content of ~8 wt.-%, the formation of blocky insertion structures leads to phase separation, with a parallel significant increase of solubility in cold xylene (XCS). SSCs offer a clear advantage here, reducing the reactivity difference and giving access to a much wider range of ethylene content and crystallinity [13]. The reduced crystallinity is paralleled by a lower glass transition temperature (*T*_g_), lower stiffness and higher transparency, the latter two being closely linked to reduced lamellar thickness and spherulitic size. 1-Butene was originally only used in terpolymers with propylene and ethylene, as for ZNC the lower reactivity limits the incorporation of 1-butene alone. Pure propylene-1-butene random copolymers are more easily accessible with SSC, offering a unique property combination. A good combination of stiffness and transparency with low XCS content results from the partial incorporation of the 1-butene unit in the crystal lamellae [14,15], but this is detrimental for ductility and *T*_g_. Random co- and terpolymers with 1-hexene have been studied increasingly in recent years, again as a result of their easier accessibility through SSC-based polymerization [16,17,18,19,20]. The longer comonomer unit changes the nature of the copolymers increasingly towards ductility, and especially at higher content the observed δ-modification goes together with good transparency and the absence of necking in tensile tests [16,19]. Still, a low amount of extractable fraction can be combined with reduced glass transition temperature at lower 1-hexene content [15].

Two further design parameters are highly relevant for industrial practice: external nucleation through the addition of selective nucleating agents [21,22,23], and the variation of processing conditions [8,9,14,20]. While the latter depends to a large degree on the final application, respectively the type of processing, and can hardly be controlled independently, the former makes it possible to balance the properties to some extent. Especially, propylene-ethylene random copolymers have been shown to affect the efficiency of specific nucleating agents for the α-modification [9,24] and the β-modification [22,24] of iPP significantly, with an increasing number of chain defects promoting the formation of γ-modification but preventing the same for β-modification.

The interaction between polymer type, nucleation and cooling rate in terms of property control has been discussed by our group before, both in a more general way [9] and for the specific case of heterophasic ethylene-propylene copolymers [25]. In the present paper, this is done for random copolymers, considering catalyst-related regio-defects and comonomer type with ethylene and 1-hexene copolymers. Additionally, a soluble nucleating agent of the sorbitol type was used to modify the behavior.

## 2. Materials and Methods

### 2.1. Materials

Homopolymers and random copolymers of propylene with ethylene or 1-hexene were analyzed based on both metallocene and Ziegler–Natta catalysts. All polymers were produced in a bench-scale reactor in a single-step polymerization in liquid propylene bulk. A supported metallocene catalyst as described for example by Boragno et al. [26] was used to prepare propylene copolymers with 3 wt.-% ethylene and 3 and 5 wt.-% 1-hexene. For the sake of comparison, polypropylenes with 0, 2.2 and 4 wt.-% ethylene were prepared using a Ziegler–Natta catalyst with a non-phthalate internal donor developed by Borealis [27].

Details about comonomer content, melt flow rate (MFR at 230 °C under 2.16 kg) and melting temperature (*T*_m_) of the non-nucleated compositions are presented in Table 1. Table 2 shows the corresponding data for the nucleated compositions. The SSC- and ZNC-based homopolymers have already been analyzed in previous studies, where the amount of 2,1 regio-defects was reported for the SSC-based iPP of 0.7% [28]. We assumed that the SSC-based random copolymers had a similar amount of regio-defects since the same catalyst system was used. Confirmation in literature can be found in cases where identical catalyst in copolymerization for comonomer contents up to about 10 mol.-% was used [13]. The polydispersity of the SSC-based preparations was determined to about 4–4.5 by high-temperature size-exclusion chromatography (SEC), being in a similar range for the ZNC-based polymers as these have been modified by visbreaking with peroxide. After polymerization, the polymer powder was stabilized in a laboratory twin-screw extruder (PRISM TSE 24, Thermo Electron Corp., Staffordshire, UK), applying a temperature profile from hopper to die of 170–190–210–220–200 °C, throughput of 2.5 kg/h, and a screw speed of 180 rpm. We used a synergistic blend of the stabilizers Irgafos 168 (CAS No. 31570-04-4) and Irganox 1010 (CAS No. 6683-19-8), which are commercially available from BASF SE, Ludwigshafen, Germany. The compositions in Table 2 were nucleated with the soluble nucleating agent 1,3:2,4-bis(3,4-dimethylbenzylidene)sorbitol (DMDBS, commercially available as Millad 3988 of Milliken Chemical, Ghent, Belgium) from the sorbitol family.

The film-gated injection-molded specimens for haze and flexural properties were molded on an Engel victory 60 Tech injection molding machine (Engel, Schwertberg, Austria), equipped with a small (Ø 22 mm; L/D = 20) screw according to EN ISO 1873-2. The processing parameters are shown in Table 3.

### 2.2. Methods

Fast scanning chip calorimetry (FSC) was done using a power-compensation Flash DSC 1 instrument from Mettler-Toledo. Samples were prepared by microtoming to obtain thin sections with a thickness of about 10 µm, followed by a reduction of their lateral size to 50–100 µm using a knife and a stereomicroscope. Before loading the sample onto the sensor, the latter was conditioned and temperature-corrected according to the instrument-provider recommendations. In addition, a thin layer of Wacker silicon oil AK 60,000 was spread on the sensor in order to improve the thermal contact between sensor and sample.

FSC was employed to perform non-isothermal crystallization and heating experiments, in particular to gain information about solidification of the melt at rapid-cooling conditions and at high supercooling of the melt, as in processing.

**Figure 1:**
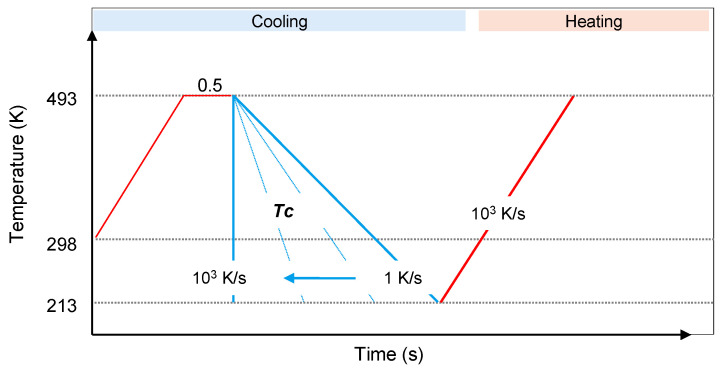
Temperature–time profile used in the analysis of structure formation at different cooling rates. The final, red-colored heating segment served for analysis of the fraction of crystals formed in the prior cooling step.

Non-isothermal crystallization experiments were performed using the temperature–time profile shown in Figure 1. The samples were heated to 493 K, equilibrated at this temperature for 0.5 s and then cooled at different rates between 1 and 1000 K/s to 213 K. Subsequently, the samples were heated to 493 K using a rate of 1000 K/s. The cooling scans were analyzed regarding the crystallization temperature, and the heating scans were inspected regarding the occurrence of a cold-crystallization event, indicating incomplete crystallization during prior cooling.

### 2.3. Mechanical and Optical Properties

Flexural modulus was determined in 3-point-bending mode on a Zwick B-Polar machine (ZwickRoell S.r.l., Genova, Italy) according to ISO 178. Injection-molded specimens with dimensions 80 × 10 × 4 mm^3^ were tested. The specimens were kept for 96 h at room temperature before testing.

Haze was measured according to ASTM D 1003 on 60 × 60 × 1 mm^3^ on injection-molded plaques using a Haze Gard Plus hazemeter (BYK-Gardner GmbH, Columbia, MD, USA).

## 3. Results

### 3.1. Non-Isothermal Crystallization

**Figure 2:**
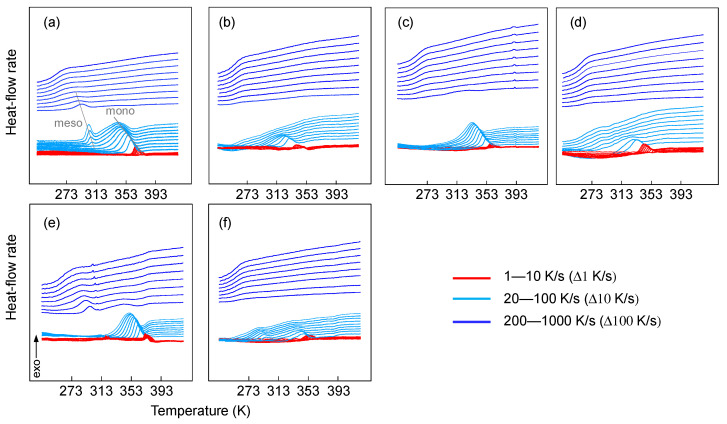
Heat-flow rate data collected on cooling of propylene homopolymers and random copolymers based on SSC (**top**) and ZNC (**bottom**) at different rates, as indicated. (**a**) SSC.iPP-0; (**b**) SSC.iPP.Eth-3; (**c**) SSC.iPP.Hex-3; (**d**) SSC.iPP.Hex-5; (**e**) ZN.iPP-0; (**f**) ZN.iPP.Eth-4.

Figure 2 shows heat-flow data collected upon cooling at different rates of isotactic polypropylene (iPP), random propylene-ethylene (iPP-Eth) and propylene-1-hexene (iPP-Hex) copolymers. The abbreviations SSC and ZN indicate the catalyst type used to synthesize the corresponding polymer. The data were collected by FSC on cooling the melt at rates from 1 to 1000 K/s. Two separate ordering processes were observed at different temperatures, as noted with “mono” and “meso”. The high-temperature peak (mono) is typically associated to the crystallization of the stable monoclinic polymorph of iPP, while the low-temperature peak (meso) is due to the formation of mesophase [20,29]. The high-temperature and low-temperature crystallization events are associated with heterogeneous and homogeneous crystal nucleation, respectively, which exhibit largely different kinetics and temperature dependencies. Detailed information about the thermodynamics of the different nucleation mechanisms is provided elsewhere [8,14,15,30]. In the case of the ZN.iPP homopolymer, cooling at rates up to 100 K/s triggered the formation of a single exothermic peak at high temperatures related to crystallization of the α-phase. Solidification at rates between 100 and 300 K/s also allowed the formation of mesophase, evidenced by the low-temperature exothermic peak. Cooling the quiescent melt at rates higher than 300 K/s demonstrated exclusive mesophase formation, which was suppressed if the cooling was performed at rates faster than 500 K/s. In the case of the SSC.iPP homopolymer, the low-temperature peak was detected at slower cooling rates (i.e., 60 K/s). If the cooling rate exceeded 100 K/s, then the high-temperature crystallization was replaced by mesophase formation occurring at the low temperature. Finally, cooling at rates faster than 300 K/s led to complete vitrification of the supercooled melt at the glass transition temperature of about 260–270 K. The presence of ethylene or 1-hexene co-units in the propylene chain shifted the critical cooling rate for the suppression of crystallization to lower rates as a function of the concentration of the co-units. Copolymers with 3 wt.-% of ethylene or 5 wt.-% of 1-hexene did not crystallize if the cooling rate exceeded 60 K/s. In the case of SSC.iPP-Eth.3 and SSC.iPP-Hex.3, a single exothermic event was recorded, which broadened at higher cooling rates. It can be suggested that crystallization and mesophase formation overlapped at the highest cooling rates, which resulted in a single broad exothermic peak [20].

While the above-described observations obtained on the iPP homopolymer and copolymers confirm earlier research in this field, the experiments in this study compared the effects of different catalyst systems on the structure formation of propylene homo- and copolymers.

**Figure 3:**
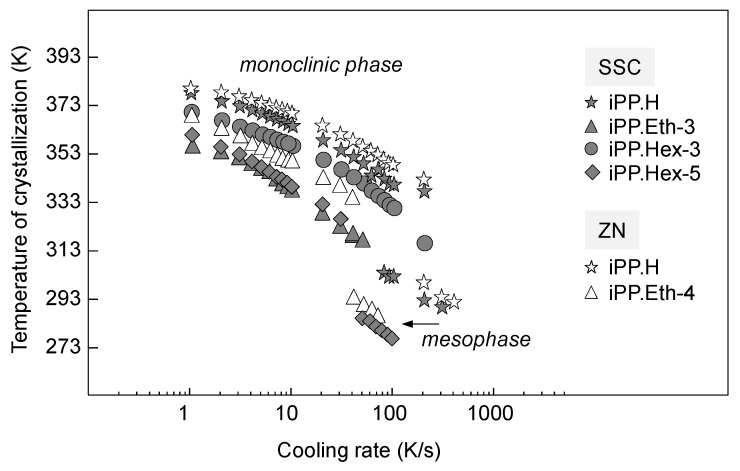
Temperature of crystallization as a function of cooling rate for homopolymers and random copolymers of propylene based on either SSC or ZN catalysts.

Quantitative information about the cooling-rate dependence of the temperature is provided in Figure 3, showing the peak temperature of the phase transitions as a function of the logarithm of the cooling rate. The open and filled symbols represent data obtained on ZNC- and SSC-based preparations, respectively. Examination of the crystallization temperature shows that incorporation of ethylene or 1-hexene co-units in the iPP chain caused a decrease of the crystallization temperature in comparison to the iPP homopolymers. In addition, it can be recognized that the SSC.iPP homopolymer crystallized at lower temperatures in comparison to the ZN.iPP.

The data in Figure 3 show that besides the crystallization temperature, the insertion of comonomers or the presence of regio-defects in the iPP similarly affected the temperature of mesophase formation. At identical cooling rates, mesophase formation of the copolymers was shifted to lower temperatures in comparison to the iPP homopolymers. Alternatively, mesophase formation can be discussed as being shifted to a lower cooling rate, probably because crystallization at higher temperatures was already inhibited at lower cooling rates.

The decrease of the crystallization temperature with the inclusion of comonomer units or regio-defects may be caused by several factors. Assuming the exclusion of co-units from crystallization, the equilibrium melting temperature will be depressed due to the changed chemical potential of the liquid phase, with the corresponding melting-point depression described by the Flory equation [31]. Alternatively, co-units may be included into the crystalline phase to different degrees, lowering the equilibrium melting point according to the energy of the crystal defect included [32].

In addition, besides equilibrium considerations, the segregation of molecular segments at the crystal growth front slows down the crystallization process and therefore shifts the crystallization temperature in non-isothermal experiments to lower temperatures [33]. Though the majority of studies in the field of crystallization of random copolymers with 1-hexene suggest the exclusion of these co-units from crystallization, unequivocal results do not seem to be available [16,34]. In particular, a study of the unit-cell parameters as a function of the concentration of 1-hexene co-units in iPP by de Rosa [19] seems to evidence that these defects are partially included into the monoclinic crystals. This notwithstanding, a decrease of the crystallization temperature due to random copolymerization is expected in both cases, that is, the exclusion and inclusion of co-units into the ordered phase.

**Figure 4:**
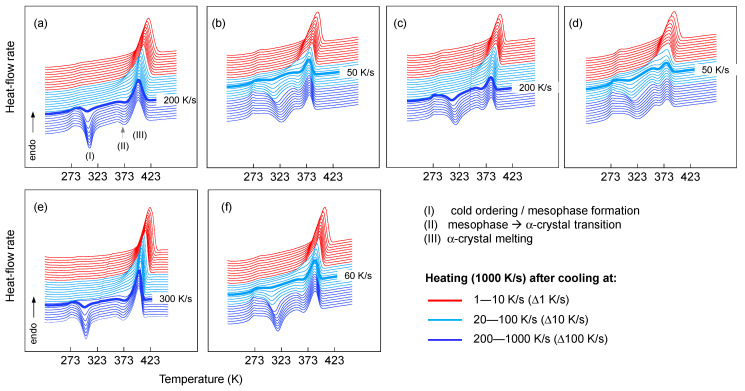
Heat-flow rate data of iPP, iPP.Eth and iPP.Hex copolymers based on SSC (**top**) and ZN (**bottom**) catalysts as a function of temperature, measured upon heating samples at 1000 K/s that were previously cooled at different rates. Bold curves indicate prior cooling at rates as indicated at the right-hand side of each curve. (**a**) SSC.iPP-0; (**b**) SSC.iPP.Eth-3; (**c**) SSC.iPP.Hex-3; (**d**) SSC.iPP.Hex-5; (**e**) ZN.iPP-0; (**f**) ZN.iPP.Eth-4.

In the present study, we analyzed the stability of the ordered phases as they were produced by cooling at different rates. The subsequent analysis of the stability and reorganization of the ordered phase was performed on the heating of samples previously cooled at different rates. Figure 4 shows the heating curves of iPP homopolymer and random copolymers after prior cooling of the melt at different rates varying from 1 to 1000 K/s. The data demonstrate the different crystallization and melting behaviors of homopolymers and random copolymers of iPP using different catalyst systems. In the case of the iPP homopolymers, fast cooling (faster than 500 K/s) suppressed crystallization and mesophase formation (dark blue curves in Figure 2). Subsequent heating of these preparations to above the glass transition temperature (dark blue curves in Figure 4) first caused exothermic mesophase formation around room temperature (I), followed by exothermic transformation of the mesophase into α-crystals at about 350−360 K (II) and finally endothermic melting of the crystals formed on heating (III).

Details of the various transitions are reported in the literature and are not discussed here because they are outside of the scope of the present work [8,35,36]. Cooling in the range of 100 to 500 K/s allowed crystallization and ordering; on subsequent heating, cold-crystallization/mesophase formation at around room temperature was reduced or absent (light blue curves in Figure 4). Slower cooling from 100 to 1 K/s increasingly permitted the completion of crystallization during cooling; on heating, only the melting of α-crystals formed during cooling was detected (red curves in Figure 4).

The heating scans of the SSC-based random copolymers reveal the same type of transitions as in the iPP homopolymers. The bold lines in each graph of Figure 4 represent the heating curves in which cold crystallization was detected for the first time after the sample was cooled at the rate shown to the right-hand side of the figure. In case of iPP.Eth-3 and iPP.Hex-5 samples, cooling at 50 K/s caused crystallization and ordering, which on subsequent heating resulted in the first detection of cold crystallization. The presence of 3 wt.-% of 1-hexene instead allowed crystallization at cooling rates up to 200 K/s, as evidenced by the subsequent heating experiment where no cold crystallization was detected.

Comparison of the heating scans collected on SSC- and ZNC-based systems revealed slightly faster crystallization kinetics in case of the ZNC polymers. The cold crystallization peak formed upon heating the ZN.iPP-0 sample was detected after cooling at 300 K/s, while in the case of SSC.iPP-0 it was already visible when cooling at 200 K/s.

**Figure 5:**
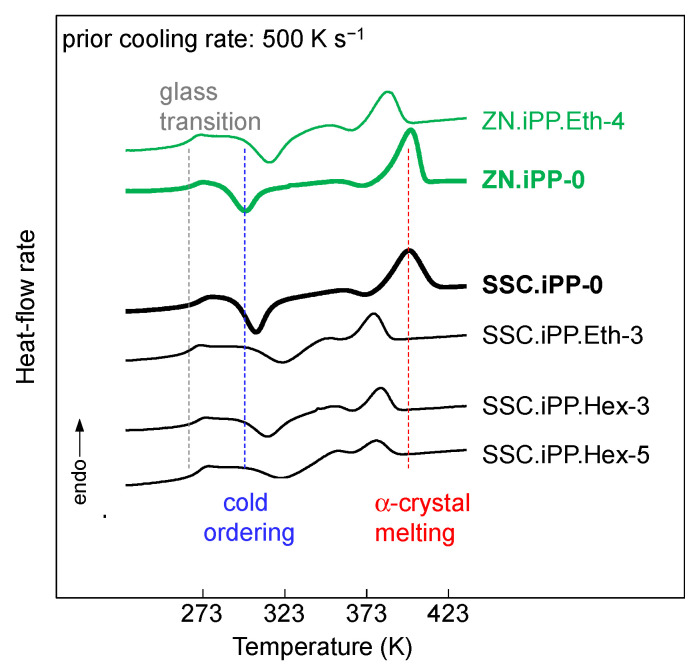
Heat-flow rate as a function of temperature collected on heating ZNC-based (green) and SSC-based (black) polypropylenes, cooled at 500 K/s. The vertical dashed lines indicate phase transitions of the ZN.iPP-0 homopolymer.

Qualitative examination of the data revealed that the SSC.iPP-0 had a higher temperature of cold crystallization/ordering in comparison to the ZN.iPP-0 sample. Figure 5 demonstrates the heat-flow rate data as a function of temperature collected on heating ZNC-based and SSC-based polypropylenes that were previously cooled at 500 K/s. The vertical dashed lines represent the various phase transitions of the ZN.iPP homopolymer. The curves marked in bold represent the homopolymers with their corresponding abbreviations shown at the right-hand side in Figure 5.

The cold ordering of the SSC.iPP homopolymer was shifted towards higher temperatures in comparison to the ZN.iPP preparation. We further observed a deceleration of the rate of mesophase formation upon heating due to the presence of co-units, which resulted in the increase of the temperature of cold crystallization (see the bottom set of curves). This increase was more pronounced for the SSC.iPP-Eth sample than for the iPP.Hex preparations. Finally, the data show that the presence of regio-defects and/or comonomer units in the iPP affected the temperature of cold crystallization and final melting of crystals, formed by reorganization of the mesophase. The temperature of the endothermic peak (III) decreased with increasing amount of co-units and regio-inclusions, which might be due to the increasing suppression of perfection and/or reorganization of the mesophase during heating. In addition, it might be suggested that the decrease of the temperature of melting was caused by the partial inclusion of ethylene or 1-hexene co-units into the mesophase. Similar observations have been made for copolymers of propylene with 1-butene [37]. X-ray analyses revealed the incorporation of 1-butene co-units into the crystalline phase and into the mesophase. In this particular study, it was suggested that the decrease of the (gross) rate of crystallization and rate of mesophase formation in propylene—butene copolymers, compared to the iPP homopolymer, was mainly due to the decrease of the thermodynamic driving force.

The data in the present study provide new insights about the kinetics of non-isothermal mesophase formation on heating influenced by either regio- or constitutional defects in iPP. It has clearly been demonstrated that the ethylene or 1-hexene co-units affected the kinetics of cold-ordering at 1000 K/s. In order to provide more information about the partitioning/exclusion of the co-units into the crystalline or mesophase, further X-ray analysis needs to be performed.

### 3.2. Nucleation Effects

**Figure 6:**
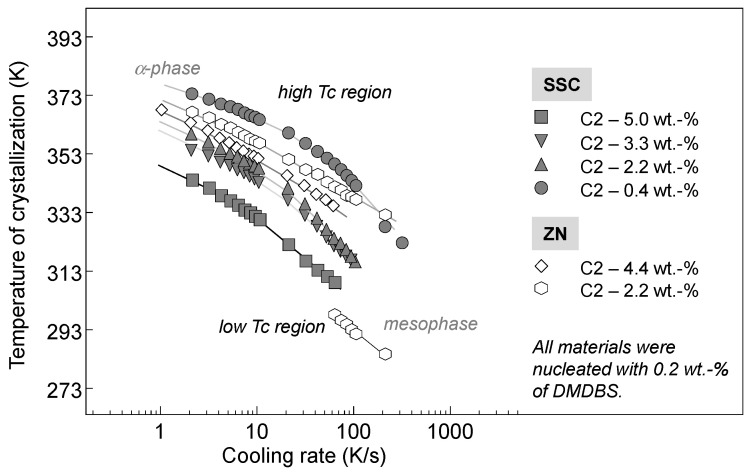
Temperature of crystallization as a function of the cooling rate of nucleated random copolymers of propylene with different amounts of ethylene based on SSC or ZN catalysts.

Figure 6 shows the temperature of crystallization as a function of the cooling rate of nucleated with DMDBS propylene—ethylene copolymers based on SSC (filled symbols) or ZN (open symbols) catalysts. DMDBS serves as a nucleating agent by the formation of a nanofibrillar network. The large surface area of the specific nanoscale network spanning the polymer melt results in the massive nucleation of small polymer crystals. DMDBS belongs to one of the most successful groups of sorbitol-based clarifying agents used to optimize the optical performance of semi-crystalline polypropylenes.

In the present study, the ethylene content of the nucleated samples was varied from 0.4 to 5 wt.-% as indicated at the right hand-side in Figure 6. The nucleated samples crystallized at relatively high temperatures at rates up to 100 K/s related to the stable monoclinic phase of iPP. At this stage, it can be assumed that the gamma-phase of iPP could also be formed. Numerous studies have shown the crystallization of gamma-phase in presence of regio- or constitutional defects at low supercoolings of non-nucleated polypropylenes [9,16]. An increase of the ethylene amount led to a shift of the crystallization to lower temperatures. The highest crystallization temperature was observed for the propylene copolymer with 0.4 wt.-% ethylene. The addition of 5 wt.-% ethylene caused an almost 20−25 K decrease in the crystallization temperature.

At similar concentrations of ethylene, ZNC-based copolymers showed slightly higher crystallization temperatures than the SSC-based copolymers. As mentioned above, due to the regio-defects present in the SSC-based copolymers, the crystallization kinetics slowed down.

### 3.3. Mechanical and Optical Properties

The random incorporation of co-units in the iPP leads to a reduced crystallization speed and temperature, resulting in the formation of an ordered phase with reduced lateral dimensions and decreased overall degree of crystallinity, which affects the final material properties [38,39,40]. This is a very important design feature for transparent and flexible films, thin-wall injection-molded articles with see-through clarity and pipes with increased toughness.

Various studies have focused on the effect of the crystal size and geometry (lamellae or globular nodules) on the tensile and optical properties in films with thickness of about 50 to 100 µm. Such thin polymer films are typically composed of the same type of crystals or ordered phase, which is not the case of injection-molded specimens. During injection molding, the semicrystalline microstructure forms under shear and thermal gradients, typically leading to the development of variable morphologies between the skin and core, with subsequent implications on the property profile [41]. It has been shown that the weight-average molecular weight (M_W_), molecular weight distribution (MWD) and addition of ethylene via copolymerization all influence the thickness of the oriented shear layer, the crystallinity, the type and amount of crystal phases and the lamellar thickness in injection-molded specimens [42].

**Figure 7:**
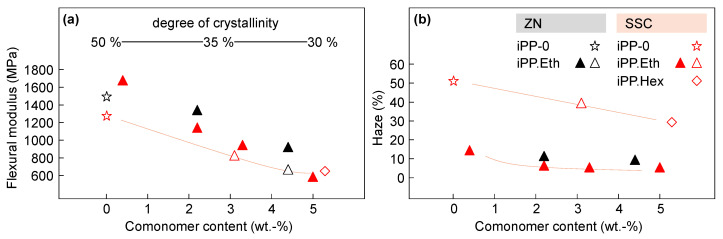
Flexural modulus of elasticity (**a**) and haze (**b**) as a function of comonomer content of propylene random copolymers based on SSC or ZN catalysts. Filled symbols stand for nucleated samples and non-filled symbols for non-nucleated samples.

Figure 7 shows the flexural modulus of elasticity (left) and the haze (right) as a function of the comonomer content for ZNC- and SSC-based polypropylene systems. In addition, the degree of crystallinity is presented in the top-left plot of Figure 7. The data were collected on nucleated (filled symbols) and non-nucleated (non-filled symbols) samples.

An increase of the comonomer content caused reductions in crystallinity, flexural modulus and haze. The presence of nucleating agent yielded a slight change in the flexural modulus and a significant decrease in haze in the iPP.Eth copolymers. In all cases, the SSC-based copolymers showed slightly lower haze and flexural modulus than the ZNC-based materials. It is suggested that the observed differences in the presence of comonomer units were related to the degree of crystallinity, which decreased with increasing comonomer content. Besides the overall degree of crystallinity, it is important to understand the thickness distribution of the skin–core layers, their polymorphic structure and crystal size, which will be part of a future study.

## 4. Conclusions and Outlook

Random copolymers of polypropylene are a large class of commercially relevant materials showing a single-phase structure and, in comparison to iPP homopolymers, a reduced crystallinity. While in case of ZNC-based copolymers nearly all chain disturbances reducing crystallization speed result from comonomer incorporation, metallocene-based copolymers combine regio-defects and comonomers, both enhancing the inherent polymorphism of iPP. This is highly relevant for industrial application, since several of the crystal modifications offer specific properties, frequently in connection with nucleation. In the present study, quantitative analysis of the kinetics of ordering processes under non-isothermal conditions revealed a strong influence of the presence of 1-hexene co-units at concentrations higher than 3 wt.-% and regio-defects in the case of the SSC-based polypropylenes. As such, we observed that the cooling rate required for the replacement of crystals by mesophase decreased significantly by about one order of magnitude from 100–200 K/s for the iPP to 50 K/s in the case of ethylene comonomer and 20 K/s in the case of SSC.iPP-Hex.5, respectively. The ZNC-based preparations showed higher temperature of crystallization and mesophase formation at identical cooling rates. The nucleation of propylene—ethylene copolymers extended the range of cooling rates to obtain semicrystalline samples from 50 K/s for non-nucleated preparations to 300−400 K/s for nucleated ones.

In order to confirm the presence of α-crystals/mesophase and their fraction as a function of the cooling rate, X-ray analysis needs to be performed as a next step of this study. Furthermore, analysis by nuclear magnetic resonance (NMR) could help to obtain a deeper understanding about the distribution of the regio-defects and co-units in polypropylene and correlate it to the final properties. It might explain the peculiar behavior of the SSC.iPP-Hex.3 sample, which showed faster crystallization and mesophase formation than the propylene–ethylene copolymer. Overall, metallocene-based random copolymers offer new property combinations, especially by achieving lower extractable fractions and melting temperatures for better application performance, for example, in advanced food packaging solutions. Substituting the mostly used ethylene by 1-hexene as comonomer further expands the accessible property range, and this can also be realized more effectively with metallocene catalysts.

## Figures and Tables

**Table 1:** Molecular characteristics of the non-nucleated samples of the present study.

Polymer Code	C2 (wt.-%)	C6 (wt.-%)	*T*_m_^1^ (°C)	MFR ^2^ (g/(10 min))
SSC.iPP-0	0		154	8.0
SSC.iPP.Eth-3	2.6		-	1.8
SSC.iPP.Hex-3		3.0	137	1.5
SSC.iPP.Hex-5		5.3	135	1.5
ZN.iPP-0	0		165	8.0
ZN.iPP.Eth-4	4.4		140	2.0

^1^*T*_m_ = melting temperature determined by differential scanning calorimeter on heating at 10 K/min. ^2^ MFR = melt-flow rate (230 °C, 2.16 kg).

**Table 2:** Molecular characteristics of the nucleated samples of the present study.

Polymer Code	C2 (wt.-%)	*T*_m_^1^ (°C)	MFR ^2^ (g/(10 min))
SSC.iPP.Eth-04-Nu	0.4	155	1.5
SSC.iPP.Eth-2-Nu	2.2	130	1.3
SSC.iPP.Eth-3-Nu	3.3	125	1.8
SSC.iPP.Eth-5-Nu	5.0	112	1.2
ZN.iPP.Eth-4-Nu	4.4	145	1.7
ZN.iPP.Eth-2-Nu	2.2	152	2.2

^1^*T*_m_ = melting temperature determined by differential scanning calorimeter on heating at 10 K/min. ^2^ MFR = melt-flow rate (230 °C, 2.16 kg).

**Table 3:** Injection-molding parameters for producing specimen.

Parameters, Units	80 × 10 × 4 mm^3^	60 × 60 × 1 mm^3^
Melt temperature, °C	230	230
Tool temperature, °C	40	40
Holding pressure time, s	40	40
Flow front speed, mm/s	206	56
Cooling time, s	15	15
